# Oligomerization and Ca^2+^/calmodulin control binding of the ER Ca^2+^-sensors STIM1 and STIM2 to plasma membrane lipids

**DOI:** 10.1042/BSR20130089

**Published:** 2013-10-31

**Authors:** Rajesh Bhardwaj, Hans-Michael Müller, Walter Nickel, Matthias Seedorf

**Affiliations:** *Zentrum für Molekulare Biologie der Universität Heidelberg (ZMBH), DKFZ-ZMBH Alliance, University of Heidelberg, Im Neuenheimer Feld 282, 69120 Heidelberg, Germany; †Heidelberg University Biochemistry Center, Im Neuenheimer Feld 328, 69120 Heidelberg, Germany

**Keywords:** calmodulin (CaM), ER–PM contact site, phosphoinositides, K-rich domain, STIM2, store-operated calcium entry (SOCE), Ca^2+^, calcium, CAD, channel-activation domain, CaM, calmodulin, CC, coiled-coil, CTD, C-terminal domain, DTT, dithiothreitol, ER, endoplasmic reticulum, GFP, green fluorescent protein, KPi, potassium phosphate, NCBI, National Center for Biotechnology Information, n.s., not significant, PC, phosphatidylcholine, PM, plasma membrane, SAM, sterile alpha motif, SDM, site-directed mutagenesis, SOCE, store-operated calcium entry, STIM, stromal interaction molecule

## Abstract

Ca^2+^ (calcium) homoeostasis and signalling rely on physical contacts between Ca^2+^ sensors in the ER (endoplasmic reticulum) and Ca^2+^ channels in the PM (plasma membrane). STIM1 (stromal interaction molecule 1) and STIM2 Ca^2+^ sensors oligomerize upon Ca^2+^ depletion in the ER lumen, contact phosphoinositides at the PM via their cytosolic lysine (K)-rich domains, and activate Ca^2+^ channels. Differential sensitivities of STIM1 and STIM2 towards ER luminal Ca^2+^ have been studied but responses towards elevated cytosolic Ca^2+^ concentration and the mechanism of lipid binding remain unclear. We found that tetramerization of the STIM1 K-rich domain is necessary for efficient binding to PI(4,5)P_2_-containing PM-like liposomes consistent with an oligomerization-driven STIM1 activation. In contrast, dimerization of STIM2 K-rich domain was sufficient for lipid binding. Furthermore, the K-rich domain of STIM2, but not of STIM1, forms an amphipathic α-helix. These distinct features of the STIM2 K-rich domain cause an increased affinity for PI(4,5)P_2_, consistent with the lower activation threshold of STIM2 and a function as regulator of basal Ca^2+^ levels. Concomitant with higher affinity for PM lipids, binding of CaM (calmodulin) inhibited the interaction of the STIM2 K-rich domain with liposomes in a Ca^2+^ and PI(4,5)P_2_ concentration-dependent manner. Therefore we suggest that elevated cytosolic Ca^2+^ concentration down-regulates STIM2-mediated ER–PM contacts via CaM binding.

## INTRODUCTION

Ca^2+^ (calcium) is a universal intracellular messenger triggering key cellular functions [1]. Ca^2+^ signals are encoded by local and periodic increase of cytosolic [Ca^2+^] in response to upstream signals. This requires low cytosolic [Ca^2+^] in the range of 10–100 nM and steep Ca^2+^ gradients across the ER (endoplasmic reticulum) and PM (plasma membrane), which are maintained by Ca^2+^ pumps [1]. Signal-dependent activation of Ca^2+^ channels in the ER and PM results in Ca^2+^ influx and elicits a variety of signals.

The predominantly ER-localized protein STIM1 (stromal interaction molecule 1) controls the opening of PM Ca^2+^ channels in a process known as store-operated Ca^2+^ entry [SOCE (store-operated calcium entry)] pathway [2]. STIM1 and the closely related STIM2 are single spanning type I membrane proteins, each having a Ca^2+^-binding EF-hand domain exposed to the ER lumen. Dissociation of Ca^2+^ from the EF-hand domains triggers aggregation via SAM (sterile alpha motif) domains [3,4]. CC (coiled-coil) domains in the cytosol-exposed C-termini of STIM1 and STIM2 propagate this oligomerization, which results in binding to PM lipids via a lysine (K)-rich domain [5–8] and in a transition from a closed to an extended conformation [9,10]. This conformational change leads to binding of the CAD (channel-activation domain) of STIM1 and STIM2 to Ca^2+^ channels in the PM, such as Orai1, resulting in channel opening [7,10–13].

STIM2 evolved during early chordate evolution as result of gene duplication [14]. The EF-hand domain of STIM2 has lower affinity for Ca^2+^ compared with STIM1 and its translocation to ER–PM contact sites occurs upon smaller decreases of ER [Ca^2+^], consistent with a function of STIM2 in regulation of basal [Ca^2+^] [15,16]. Also, consistent with this lower activation threshold, the CTD (C-terminal domain) of STIM2 has a higher affinity to PI(4,5)P_2_-containing liposomes as compared with STIM1, as shown by *in vitro* liposome binding experiments [6]. This higher affinity for PM lipids may help to position STIM2-containing ER at the PM, which on one hand sensitizes the activation of STIM2 in response to minor decreases in luminal [Ca^2+^] but on the other hand exposes cells to the risk of Ca^2+^ overload.

Cytosolic Ca^2+^ can also contribute to the regulation of SOCE. For example the cytosolic Ca^2+^-sensing protein CRACR2A regulates the interaction of STIM1 and Orai1 [17] and Ca^2+^-loaded CaM (calmodulin) binds to cytosol-exposed N- and C-termini of Orai1 resulting in a Ca^2+^-dependent inactivation of the channel [18,19]. Since STIM1 and STIM2 comprise several predicted Ca^2+^/CaM-binding sites in their CTDs [20], cytosolic Ca^2+^ may also contribute directly to their regulation. One of the Ca^2+^/CaM-binding sites overlaps with the lipid-binding K-rich domain of STIM1 and STIM2 and isothermal titration calorimetry with STIM1 and STIM2 peptides revealed *K*_d_'s for Ca^2+^/CaM of 0.8 and 0.9 μM, respectively [21]. This suggests that binding of Ca^2+^/CaM may compete with lipid binding and ER–PM contact formation but the mechanism of this interaction remains unknown.

Using *in vitro* binding studies with recombinant STIM1 and STIM2 CTDs and isolated K-rich domains, we found that binding to PI(4,5)P_2_-containing liposomes depends on the oligomerization of the K-rich domains. STIM2 K-rich domain forms an amphipathic α-helix and can dimerize, which leads to higher affinity to PI(4,5)P_2_-containing liposomes compared with the K-rich domain of STIM1. The formation of this α-helix in the STIM2 K-rich domain creates an overlapping Ca^2+^/CaM-binding site and binding of Ca^2+^/CaM interferes with binding of STIM2 to PM lipids.

## MATERIALS AND METHODS

### STIM1, STIM2 and CaM constructs

The constructs for bacterial expression of N-terminally His_(6)_–GFP (green fluorescent protein)-tagged CTDs of human STIMs were created by ligation of DNA fragments encoding STIM1 [NCBI (National Center for Biotechnology Information) Accession Number: NP_003147.2] from residues 233–685 (GFP–STIM1C) and STIM2 (NP_065911.3) from residues 237–746 (GFP–STIM2C) into SalI/BamHI restriction sites downstream of GFP in pET15b vector [6]. The K-rich domain deletion mutant of GFP–STIM1C (pEE142) was generated by SDM (site-directed mutagenesis), where a stop codon (TAG) was introduced after Gly^670^ of STIM1. The K-rich domain deletion mutant of GFP–STIM2C (pMS729) was generated by SDM, where Lys^730^ of STIM2C was mutated to a stop codon (TGA). Plasmids encoding GFP–STIM1K (pEE50) and GFP–STIM2K (pEE56) with residues 662–685 of human STIM1 and GFP–STIM2K with residues 720–746 of human STIM2 were cloned as previously described [6].

The tetramerization domain of the Gcn4 leucine zipper variant of pLI [22] was cloned into pET15b–GFP–STIM1K downstream of GFP by using SalI restriction sites to create GFP–Zipper–STIM1K (pMS677). The GFP–Zipper (pMS678) was generated by creating a stop codon (TAG) downstream of the Gcn4 zipper in the GFP–Zipper–STIM1K construct by SDM. Mutation of Pro^682^ in GFP–STIM1K to alanine by SDM created GFP–STIM1K–P682A (pMS646). Pro^675^ in both GFP–STIM1K- and GFP–STIM1K-P682A-carrying pET15b constructs was mutated to alanine (GCT) to create GFP–STIM1K–P675A (pMS680) and GFP–STIM1K–P675A–P682A (pMS681). Mutation of Lys^743^ in GFP–STIM2K to proline created GFP–STIM2K–K743P (pEE57). Leu and Cys residues were inserted after Asp^666^ in GFP–STIM1K–P675A–P682A to create GFP–STIM1K+LC–P675A–P682A (pMS781). GFP–STIM2K–C725A (pMS720) was created by mutation of Cys^725^ in GFP–STIM2K to alanine.

Human CaM and CaM1–4 mutant (CaM1–4m) were PCR amplified from pCDNA3–CFP–CaM and pCDNA3–CFP–CaM1-4-mutant [23] with 5′ NdeI and 3′ BamHI restriction sites. NdeI/BamHI DNA fragments were ligated into pET15b-His_(6)_ vector for bacterial expression of N-terminally His_(6)_-tagged human CaM (pMS644) and CaM1–4m (pMS645). CaM1–2m (pMS704) was created by sequential mutations of Asp^21^ in EF-hand I to alanine and Asp^57^ in EF-hand II to alanine. CaM3–4m (pMS705) was created similarly by SDM, where sequential mutations of Asp^94^ in EF-hand III to alanine and Asp^130^ in EF-hand IV to alanine were created. For all the SDM reactions, Pfu Turbo DNA Polymerase (Agilent Technologies) amplified DNA was digested by DpnI restriction enzyme (NEB) and amplified in *Escherichia coli*. DNA sequences of all constructs were confirmed by sequencing.

### Multiple sequence analysis

Sequences of the STIM proteins were obtained from database searches such as NCBI, UniProt and Ensembl. Sequence alignment of K-rich domains of STIM proteins from representative species was performed using Clustal Omega, followed by a minor manual refinement.

### Protein expression and purification

Recombinant GFP, GFP-fusion proteins, CaM and its Ca^2+^-binding mutants were purified from BL21 (DE3) *E. coli* using a N-terminal His_(6)_-tag. Protein expression was induced at 25°C by 0.125 mM IPTG (isopropyl β-d-thiogalactopyranoside) in 500 ml cultures of 0.5–0.6 OD_600_. Overnight-cultured cells were harvested, resuspended in freshly made lysis buffer with 50 mM Tris/HCl pH 7.5, 250 mM NaCl, 2 mM imidazole, 2 mM EDTA (except for CaM and its Ca^2+^-binding mutants), 2 mM DTT (dithiothreitol), 1 mM PMSF and 1X Complete™ (protease inhibitor cocktail, Roche) and lysed using a high-pressure homogenizer (EmulsiFlex-C5, Avestin) at 15000-20000 psi (1 psi=6.9 kPa). After ultracentrifugation (45 Ti rotor, Beckman Coulter, Inc.) of the lysate at 120000 ***g*** for 45 min at 4°C, the supernatant was incubated for 1 h at 4°C with 0.5 g Protino® Ni-TED resin (Macherey-Nagel GmbH & Co.). Protein-bound resin was washed thrice with lysis buffer and the proteins were incubated for 1 h at 4°C with 2-4 ml lysis buffer containing 300 mM imidazole for elution. The eluted proteins were then concentrated to 0.5 ml using Vivaspin® 6 centrifugal concentrators (Sartorius Stedim Biotech) of different molecular mass cut-offs (10 kDa for GFP, 30 kDa for GFP–STIMs C-termini and 5 kDa for CaM). For further purification, size-exclusion chromatography of concentrated proteins was performed by using a Superdex 200 10/300 GL column pre-equilibrated with HK buffer (25 mM HEPES/KOH pH 7.5, 150 mM KCl, 1 mM DTT) on an Äkta purifier (GE Healthcare) at a flow rate of 0.5 ml/min. Molar extinction coefficient and molecular weight of each protein was used to measure the protein concentration of eluted fractions by NanoDrop® ND-1000 spectrophotometer (Thermo Scientific). Purified proteins were divided into aliquots in low protein binding micro tubes (Sarstedt), were flash-frozen using liquid nitrogen and stored at −80°C.

### Non-reducing SDS/PAGE

Protein samples dialysed overnight at 4°C against non-reducing HK buffer were incubated at 65°C for 5 min in non-reducing sample buffer [50 mM Tris/HCl, pH 6.8, 2% (w/v) SDS, 4 M urea and 10% (v/v) glycerol] and subjected to SDS/PAGE followed by 1 h of staining with 2.5% (w/v) Coomassie Brilliant Blue solution.

### FACS-based liposome-binding assay

Gel-filtration purified 1 or 2 μM GFP-tagged protein samples (100 μl) were incubated at 25°C with BSA-blocked rhodamine–phosphatidylethanolamine labelled PM-like liposomes for 2 h in HK buffer either in reducing (1 mM DTT) or non-reducing conditions. PM-like- and PC (phosphatidylcholine) liposomes with different [PI(4,5)P_2_] were made as previously described [24]. After addition of 1 ml HK buffer, the liposomes were pelleted for 10 min at 4°C at 15000 g and were then resuspended in 400 μl HK buffer. The liposomes bound to GFP-fusion proteins were detected by flow cytometry (BD FACSCalibur™) and data were processed using BD CellQuest™ Pro version 5.2 (BD Biosciences) as described [24].

### Peptide synthesis and CD spectroscopy

The peptides corresponding to residues 671–685 and 730–746 of wild-type human STIM1 (NCBI Accession Number: NP_003147.2) and STIM2 (NP_065911.3) were obtained from Peptide Specialty Laboratories GmbH. CD measurements were performed on a Jasco Corp. J-715 Spectropolarimeter at 25°C using a 0.1 cm path length quartz cuvette, with a peptide concentration of 150 μM in 5 mM potassium phosphate (KP_i_) buffer at pH 7.6.

### Detergent-free fluorescence-based CaM-binding assay

The GFP or GFP-fusion proteins (1 μM, 600 μl) were incubated with 30 μl of CaM beads (Agilent Technologies) after washing thrice with CaM-binding buffer [25 mM Tris/HCl (pH 7.5), 150 mM NaCl, 1 mM CaCl_2_ or 1 mM EGTA]. After 1 h incubation at 4°C on a rotating device, CaM beads were pelleted at 100 g for 1 min at 4°C. The beads were washed thrice with the CaM-binding buffer and incubated with 600 μl elution buffer [25 mM Tris/HCl (pH 7.5), 1 M NaCl, 4 mM EGTA] for 1 h at 4°C, while rotating. GFP fluorescence of 40 μl of each input, unbound and bound fractions was measured using a 384-well polystyrene black microplate (Greiner Bio-One GmbH) in a microplate reader (Thermo Scientific Appliskan). The amount of protein bound was calculated as percentage of input protein bound to CaM beads.

### CaM-binding assay using size-exclusion chromatography

Purified His_(6)_-tagged CaM or Ca^2+^-binding mutants of CaM (10 μM) were incubated with 10 μM GFP-tagged STIM2 K-rich domain dimer for 1 h at 4°C in a 1 mM CaCl_2_ containing non-reducing HK buffer in a reaction volume of 100 μl. Roughly 50 μl of the reaction was injected into a Superdex 75 5/150 GL column (GE Healthcare) pre-equilibrated with the same buffer and separated with a flow rate of 0.125 ml/min. The UV chromatogram of Ca^2+^/CaM in presence of GFP–STIM2K dimer at 280 nm was overlayed with chromatograms of runs from 10 μM CaM alone and GFP–STIM2K dimer alone to assess if the peak shifts towards a larger elution volume indicating complex formation. SDS/PAGE analysis of eluted fractions was performed to further assess the amount of complex formation.

### CaM- and lipid-binding competition assay

Gel-filtration purified CaM or Ca^2+^-binding mutants of CaM (5 μM) were incubated with 2 μM GFP-STIM2K dimer for 1 h at 4°C in presence of 100 μM CaCl_2_ in a non-reducing HK buffer. 100 μl of the reaction was incubated for 2 h at 25°C with 25 μl BSA-blocked PC- or PM-like, 5 or 2 mol% PI(4,5)P_2_-containing liposomes. The effect of CaM or Ca^2+^-binding mutants of CaM on lipid-binding activity of GFP–STIMs was assessed by comparing the amount of GFP-fusion proteins bound to liposomes in presence and in absence of CaM or Ca^2+^-binding mutants of CaM. The liposomes bound to GFP-fusion proteins were detected by flow cytometry.

## RESULTS

### Oligomerization of STIM1 and STIM2 cytosolic domains leads to PI(4,5)P_2_ binding

**Figure 1:**
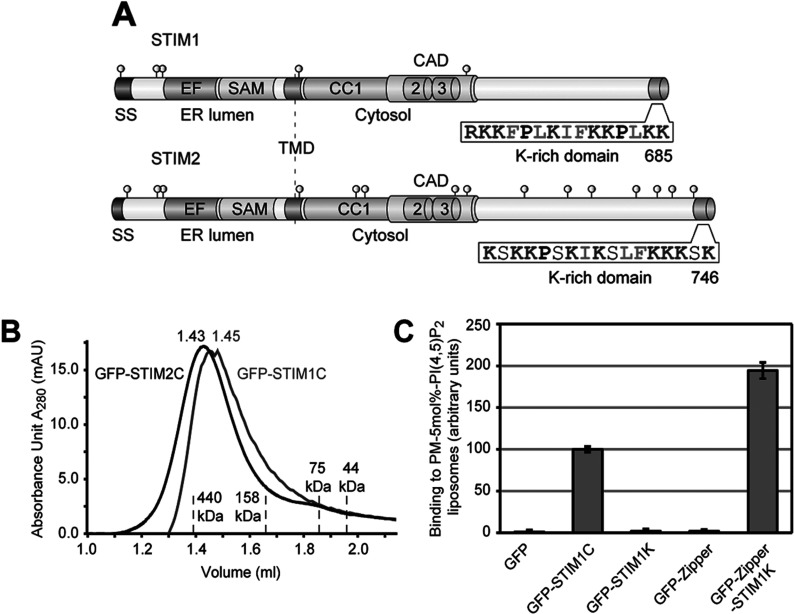
Tetramerization of STIM1 K-rich domain leads to PI(4,5)P_2_ binding (**A**) Schematic outline of human STIM1 and STIM2 domain structure (drawn to scale). SS (signal sequence), EF-hand domain (EF), SAM, TMD (transmembrane domain), CC domains 1–3, CAD, the K-rich domains with their amino acid sequences and the orientations of N- and C-termini are shown. Cysteine residues are depicted as grey circles. (**B**) Purified GFP-tagged CTDs of STIM1 and STIM2 were run on S200 gel-filtration column and the elution profiles of GFP–STIM1C (grey), GFP–STIM2C (black) and of the indicated marker proteins are shown. (**C**) Binding of 1 μM of each GFP, GFP–STIM1C, GFP–STIM1K, GFP-tagged GCN4 leucine zipper (GFP–Zipper) and GFP–Zipper with STIM1 K-rich domain (GFP–Zipper–STIM1K) to PM-like liposomes with 5 mol% PI(4,5)P_2_ in presence of 1 mM DTT. Binding of GFP–STIM1C was set to 100 and bars indicate mean±S.D. from at least three experiments.

The CTDs of STIM1 and STIM2 are exposed to the cytosol and comprise three CC domains with an overlapping CAD, variable regions and K-rich domains (Figure 1A). Binding to PI(4,5)P_2_-containing liposomes having a PM-like lipid composition with cholesterol and sphingolipids depends on electrostatic interaction of the K-rich domains with PI(4,5)P_2_ [6].

To study the mechanism by which human STIM1 and STIM2 bind lipids at the PM, we purified the CTDs of human STIM1 and STIM2 as His_(6)_–GFP-tagged proteins from *E. coli* and analysed their assembly states in solution. Gel-filtration experiments showed that both GFP-tagged CTDs of STIM1 (GFP–STIM1C) and STIM2 (GFP–STIM2C) form tetramers (Figure 1B), whereas GFP alone ran as monomer (Supplementary Figure S1; available at http://www.bioscirep.org/bsr/033/bsr033e077add.htm). In contrast, the isolated K-rich domain of STIM1 (GFP–STIM1K), uncoupled from CC-mediated oligomerization ran as monomer upon gel filtration (Supplementary Figure S1). GFP–STIM1K showed no binding to PM-like liposomes similar as free GFP, whereas tetrameric GFP–STIM1C was bound (Figure 1C). This suggests that binding of STIM1 to PI(4,5)P_2_-containing liposomes depends on oligomerization of its K-rich domain. To test this idea directly, we tetramerized STIM1 K-rich domain artificially by fusion with a leucine zipper from yeast Gcn4 transcription factor. This resulted in tetramer formation (Supplementary Figure S1) and increased lipid binding (Figure 1C). Thus, oligomerization of STIM1 K-rich domain increases the affinity for phosphoinositides at the PM.

**Figure 2:**
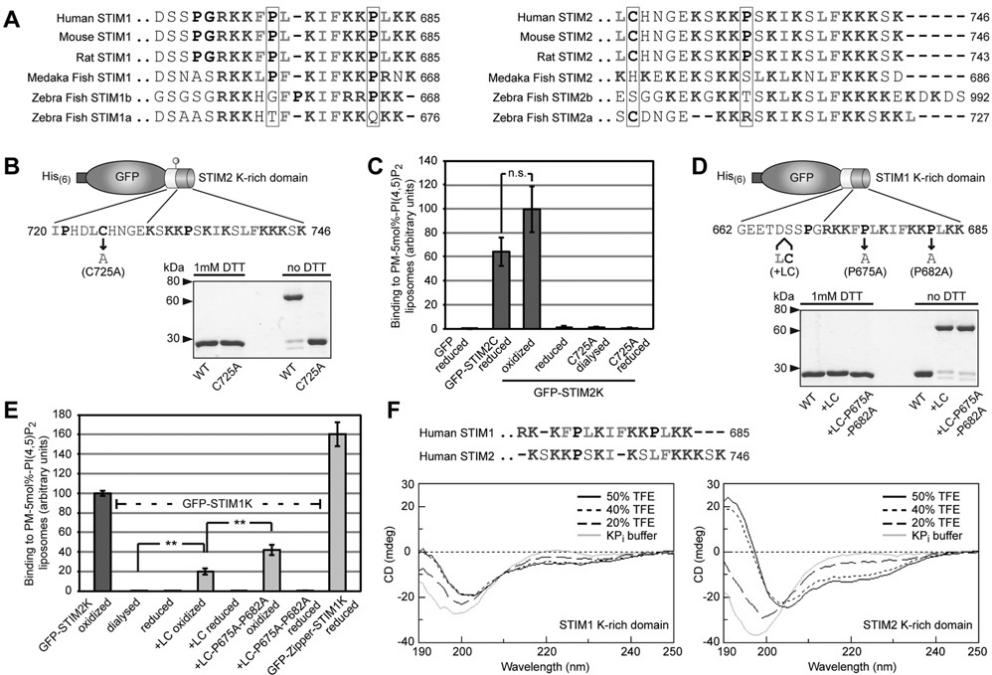
The K-rich domain of STIM2 forms an α-helix and binds PI(4,5)P_2_ as dimer (**A**) Alignment of extreme C-termini of STIM proteins from different species. Alignment of K-rich domains from STIM1 in mammals and fishes are shown on the left and STIM2 on the right. Conservation of proline and cysteine residues are highlighted (**B**) Cartoon of GFP fused to the last 27 residues of STIM2 (GFP–STIM2K) where Cys^725^ is depicted as grey circle. Coomassie Brilliant Blue stained GFP–STIM2K [WT (wild-type)] and GFP–STIM2K with a C725A mutation (C725A) after overnight dialysis in HK buffer (25 mM Hepes/KOH pH 7.5, 150 mM NaCl) and separation by SDS/PAGE under reducing and non-reducing conditions. (**C**) Binding of 1 μM reduced GFP and GFP–STIM2C, oxidized and reduced GFP–STIM2K, GFP–STIM2K–C725A dialysed against DTT-free buffer and reduced GFP–STIM2K–C725A to PM-like liposomes with 5 mol% PI(4,5)P_2_. Binding of oxidized GFP–STIM2K was set to 100. Bars indicate mean±S.D. from at least three experiments and n.s. (not significant) according to student's *t* test. (**D**) Cartoon of GFP fused to the last 24 residues of STIM1 (GFP–STIM1K) and positions of the introduced Leu and Cys residues and Pro to Ala mutations. Coomassie Brilliant Blue stained GFP–STIM1K (WT), GFP–STIM1K+LC and GFP–STIM1K+LC–P675A–P682A after overnight dialysis in HK buffer with and without 1 mM DTT and separation by SDS/PAGE under reducing and non-reducing conditions. (**E**) Binding of 1 μM oxidized GFP–STIM2K, GFP–STIM1K dialysed against DTT-free buffer, reduced GFP–STIM1K, oxidized and reduced GFP–STIM1K+LC, GFP–STIM1K+LC-P675A-P682A and reduced GFP-Zipper-STIM1K to PM-like liposomes with 5 mol% PI(4,5)P_2_. Binding of 1 μM oxidized GFP–STIM2K was set to 100. Bars indicate means±S.D. from at least three experiments (**P<0.005 according to student's *t* test). (**F**) Sequence alignment of peptides of human STIM1 (residues 671–685) and STIM2 (residues 730–746) corresponding to the K-rich domains used for CD. CD spectra of 150 μM peptide in 5 mM KP_i_ buffer pH 7.6 containing 0, 20, 40 and 50% TFE, is shown below the alignment.

Our previous studies, however, showed that the isolated K-rich domain of STIM2 (GFP–STIM2K), lacking its CC domains, can bind PI(4,5)P_2_-containing liposomes [6]. Both K-rich domains of STIM1 and STIM2 comprise a similar number of positively charged residues (eight and nine) and this is unlikely to explain the difference in lipid binding. To unravel this difference, we compared the sequences of STIM1 and STIM2 K-rich domains from different species. Most C-termini of STIM proteins in metazoa contain a K-rich sequence (Figure 2A). We also noted that unlike in STIM1, STIM2 evolved a conserved cysteine residue, which is located directly upstream of the K-rich domain. This residue may dimerize the K-rich domain during our purification procedure using dialysis under non-reducing conditions [6]. In order to test whether dimerization of STIM2 K-rich domain causes the increased affinity to PI(4,5)P_2_ liposomes, we performed overnight dialysis of GFP–STIM2K in a non-reducing (DTT-free) buffer. This led to formation of a DTT-reducible dimer, as validated by SDS/PAGE (Figure 2B). Dimerization is strictly dependent on Cys^725^ as mutation to alanine completely abolished dimer formation (Figure 2B). The STIM2 K-rich domain dimer was sufficient for binding to PI(4,5)P_2_-containing PM-like liposomes and showed no significant difference in binding compared with tetrameric STIM2 CTD in presence of DTT (Figure 2C). Only the oxidized GFP–STIM2K dimer but neither the reduced monomer nor the Cys-725-Ala mutant bound PI(4,5)P_2_ liposomes (Figure 2C).

Although, we do not know whether the CTD of STIM2 oxidizes *in vivo*, we used Cys^725^-induced STIM2 K-rich domain dimer as a tool to study the mechanism of PI(4,5)P_2_-binding. In order to compare the PI(4,5)P_2_-binding activity of the isolated K-rich domain of STIM2 with STIM1, we artificially dimerized GFP–STIM1K by introduction of a cysteine residue corresponding to the position of Cys^725^ in STIM2 (Figure 2D). Oxidation of GFP–STIM1K with a leucine and cysteine insertion after residue 666 led to 84% dimerization (Figure 2D). This dimer showed weak binding to PI(4,5)P_2_ liposomes, i.e. 5-fold less than GFP–STIM2K dimer (Figure 2E). Also the binding of GFP–STIM1K dimer was 8-fold lower than the GFP–STIM1K tetramer suggesting that tetramerization of STIM1 K-rich domain is essential for efficient lipid binding (Figure 2E).

Since dimerization of STIM1 K-rich domain did not lead to similar lipid binding as STIM2 K-rich domain dimer, we searched for additional differences in STIM1- and STIM2 K-rich sequences. After gene duplication in fishes, which resulted in *STIM1* and *STIM2* genes [14], the K-rich domains in STIM2 evolved with extreme C-termini free of proline, whereas the K-rich domains of most vertebrate STIM1 contain two conserved proline residues (Figure 2A). The lack of the terminal proline residue in mammalian STIM2 may allow the formation of a short amphipathic α-helix. Such a helix had been identified as essential feature of a similar lipid-binding signal in yeast Ist2 [25]. Ist2 functions as a ER–PM tether and introduction of a proline interferes with the localization of Ist2 in the cortical ER [25–27].

To confirm that the K-rich domain of STIM2, but not of STIM1, can form an α-helix, we analysed the secondary structures of peptides corresponding to both K-rich domains. CD showed that the STIM2 peptide formed an α-helix in the presence of trifluoroethanol, which mimics a hydrophobic membrane environment and stimulates α-helix formation [28]. A peptide corresponding to the STIM1 K-rich domain remained unstructured under these conditions (Figure 2F). To test whether this α-helix formation explains the differences in lipid binding, we mutated both prolines (Pro^675^ and Pro^682^) in the GFP-STIM1K dimer, which had no influence on dimer formation (Figure 2D). These mutations led to a 2-fold increase in PI(4,5)P_2_ binding that is 42±5% of the binding of GFP–STIM2K dimer (Figure 2E). Thus, dimerization and absence of the C-terminal proline residue in the STIM2 K-rich domain contribute to its higher lipid-binding activity.

### Co-evolution of overlapping lipid- and Ca^2+^/CaM-binding sites in STIM2

The formation of an α-helix and oxidation-driven dimerization are specific features of the STIM2 K-rich domain resulting in increased binding to PI(4,5)P_2_ at the PM. Together with a faster activation of STIM2 in response to the depletion of Ca^2+^ from the ER lumen [15], these features may contribute on the one hand to high sensitivity of STIM2 as Ca^2+^ sensor, but on the other hand they bear the risk of an overactivation of STIM2 and constitutively high cytosolic Ca^2+^ levels. In this context, the previously described Ca^2+^-dependent binding of CaM to the K-rich domains of STIM1 and STIM2 point towards an additional regulation of ER–PM contact formation via competing PI(4,5)P_2_ and Ca^2+^/CaM-binding sites [21].

**Figure 3:**
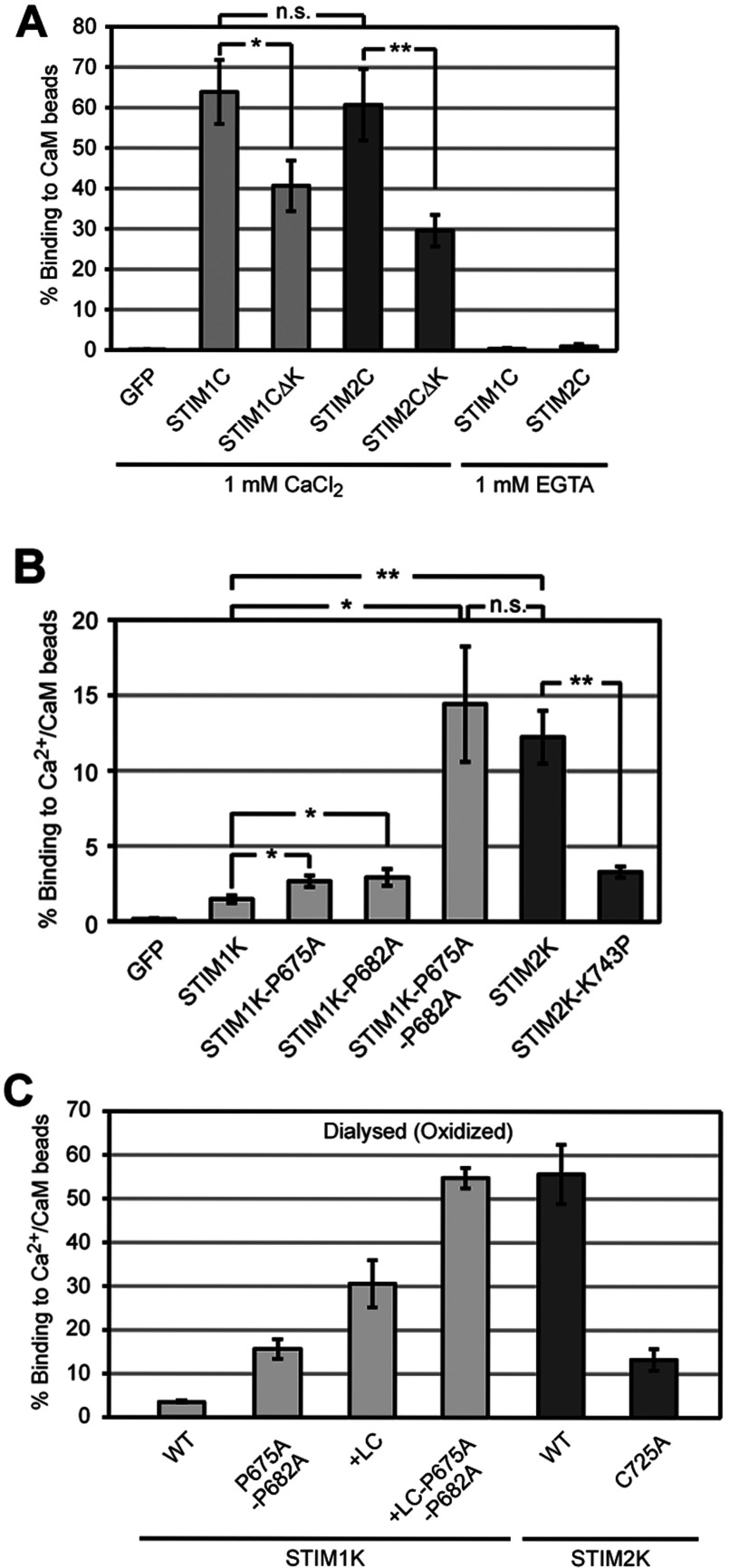
α-helicity of STIM2 K-rich domain contributes in binding to Ca^2+^/CaM (**A**) Binding of 1 μM reduced GFP, GFP–STIM1C, GFP–STIM1C∆K, GFP–STIM2C and GFP-STIM2C∆K to Ca^2+^/CaM beads in 1 mM CaCl_2_ and GFP–STIM1C and GFP-STIM2C in 1 mM EGTA. (**B**) Binding of 1 μM reduced GFP, GFP–STIM1K, GFP–STIM1K–P675A, GFP–STIM1K–P682A, GFP–STIM1K–P675A–P682A, GFP–STIM2K and GFP–STIM2K–K743P to Ca^2+^/CaM beads in 1 mM CaCl_2_. (**C**) Binding of 1 μM GFP–STIM1K, GFP–STIM1K–P675A–P682A, GFP–STIM1K+LC, GFP–STIM1K+LC–P675A–P682A, GFP–STIM2K, and GFP–STIM2K–C725A (all dialysed against DTT-free buffer) to Ca^2+^/CaM beads in 1 mM CaCl_2_. Bars indicate mean±S.D. from at least three experiments (*P<0.05, **P<0.005, n.s. according to Student's *t* test).

In order to characterize the interaction between CaM and STIM1 and STIM2 in more detail, we developed a fluorescence-based *in vitro* CaM-binding assay. Since many complexes of CaM with its targets form via hydrophobic interactions with an α-helical structure in the target [29], we established the CaM-binding assay in detergent-free buffer system. The resulting unspecific interactions were minimized by the use of reaction tubes with modified surfaces and bound proteins were eluted with high salt and the metal chelator EGTA. First, we analysed binding of the entire CTDs to CaM-beads. Under reducing conditions at 1 μM protein concentration in 1 mM CaCl_2_, 64±8% of GFP–STIM1C and 61±9% of GFP–STIM2C were bound to CaM-beads, while addition of EGTA completely abolished the binding (Figure 3A). Free GFP showed no binding, demonstrating that this assay is specific. Deletion of the K-rich domains of STIM1C and STIM2C (GFP–STIM1C∆K and GFP–STIM2C∆K) reduced the binding to Ca^2+^/CaM to 41±6 and 30±4%, respectively, suggesting these domains contribute to Ca^2+^/CaM binding (Figure 3A). Binding of GFP–STIM1C∆K and GFP–STIM2C∆K is consistent with the presence of additional Ca^2+^/CaM-binding sites, which are predicted in the CAD of STIM1 and STIM2 [20,30]. We confirmed the results from the fluorescence-based assay by analysis of GFP–STIM2C and GFP–STIM2C∆K binding to Ca^2+^/CaM by Western blotting using GFP-specific antibody and found 67±5 and 27±4% bound GFP–STIM2C and GFP–STIM2C∆K (Supplementary Figure S2; available at http://www.bioscirep.org/bsr/033/bsr033e077add.htm). This is similar to the values observed by our fluorescence-based assay. Thereafter, we employed this quantitative fluorescence-based assay for all following experiments.

In contrast to the isothermal titration calorimetry results of Bauer et al. (2008), reduced monomeric GFP–STIM2K showed 8.4-fold higher binding to Ca^2+^/CaM beads than GFP–STIM1K (Figure 3B). In order to determine whether formation of an amphipathic α-helix in STIM2 K-rich domain enhances Ca^2+^/CaM binding, we mutated both prolines in GFP–STIM1K and tested binding. Single mutations of Pro^675^ and Pro^682^ to alanine led to a 1.8- and 2-fold increase in binding, respectively, but mutation of both prolines enhanced binding to Ca^2+^/CaM 9.8-fold, i.e. to an amount similar to binding of GFP–STIM2K (Figure 3B). This confirmed that α-helicity is critical for binding of the STIM2 K-rich domain to Ca^2+^/CaM. Consistently, mutation of Lys^743^ to proline in GFP–STIM2K, to mimic the sequence of STIM1K, reduced binding 3.7-fold (Figure 3B). Since lipid binding required dimerization of GFP–STIM2K, we tested if oxidation-induced dimerization has an influence on binding Ca^2+^/CaM. GFP–STIM1K and GFP–STIM1K with both prolines of the K-rich domain mutated as well as oxidized GFP–STIM2K showed at least 3-fold increased binding to Ca^2+^/CaM compared with the reduced monomers or a GFP-STIM2K Cys-725-Ala mutant (Figure 3C). GFP–STIM1K and its double proline mutant did not show significant increase in binding to Ca^2+^/CaM upon dialysis under oxidizing conditions (Figure 3C). This enhanced binding of the oxidized dimers may result from avidity effects, assuming that multiple CaM molecules are present on one sepharose bead. Moreover, it clearly indicates that dimerization of STIM2 K-rich domain does not interfere with binding to Ca^2+^/CaM.

### Fully- and semi-saturated CaM bind STIM2 K-rich domain

**Figure 4:**
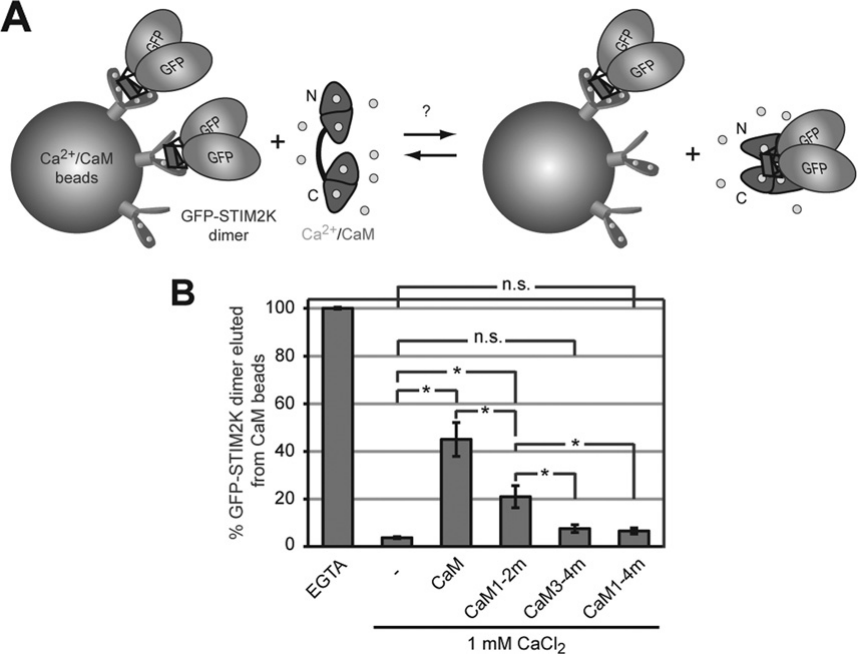
STIM2 K-rich domain binds fully- and semi-Ca^2+^-loaded CaM in solution (**A**) Cartoon with illustration of a competition assay with GFP-STIM2K bound to Ca^2+^/CaM beads and elution of GFP–STIM2K with purified CaM from the beads. (**B**) Quantification of eluted GFP–STIM2K after incubation with 4 mM EGTA, 1 mM CaCl_2_, and 75 μM CaM, CaM1-2m, CaM3–4m or CaM1-4m in 1 mM CaCl_2_. Ca^2+^-binding EF-hand domains in the N-terminal lobe (CaM1-2m), the C-terminal lobe (CaM3-4m) or in both lobes (CaM1–4m) were mutated. Bars indicate mean±S.D. from at least three experiments (*P<0.05 and n.s. according to Student's *t* test).

In order to test whether Ca^2+^/CaM and GFP–STIM2K can form a complex under conditions where GFP–STIM2K bind PI(4,5)P_2_, we performed in solution-binding experiments with different purified CaM variants and oxidized GFP–STIM2K dimer. CaM is a 17 kDa acidic cytosolic Ca^2+^-binding protein containing N- and C-terminal lobes connected by a flexible linker. Each lobe has two Ca^2+^-binding EF-hand domains [31] and since the Ca^2+^-binding affinity for the C-terminal lobe is several fold higher than for the N-terminal lobe [32,33], CaM can exist in three states: as Ca^2+^-free, semi Ca^2+^-loaded or as fully Ca^2+^-loaded molecule. In order to test under which conditions the K-rich domain of STIM2 interacts with CaM, we mutated EF-hand domains I and II in the N-terminal lobe (CaM1–2m), EF-hand domains III and IV in the C-terminal lobe (CaM3–4m), and all four EF-hand domains (CaM1–4m). Correct folding of the purified proteins was confirmed by trypsin digestion [34], where fully Ca^2+^-loaded CaM and C-terminal lobe Ca^2+^-loaded CaM (CaM1–2m) showed increased protease protection in the presence of CaCl_2_ (Supplementary Figures S3A and S3C; available at http://www.bioscirep.org/bsr/033/bsr033e077add.htm) compared with the unloaded CaM1–4m and the N-terminal lobe Ca^2+^-loaded CaM (CaM3–4m) (Figures S3B and S3D). To compare the binding of GFP–STIM2K dimer to semi and fully Ca^2+^-loaded CaM, we performed a competition experiment with GFP–STIM2K bound to Ca^2+^/CaM beads (cartoon Figure 4A). Incubation of immobilized GFP–STIM2K dimer with purified CaM in CaCl_2_ buffer eluted 45±7% of GFP–STIM2K from the Ca^2+^/CaM beads compared with only 7±1 and 8±2% elution after incubation with CaM1–4m and CaM3–4m, respectively (Figure 4B). This showed that Ca^2+^ loading of CaM is required for binding to STIM2 K-rich domain and that mutation of the EF-hand domains in the C-terminal lobe abolished binding. We confirmed these data by in solution-binding experiments under conditions that allow binding of GFP–STIM2K dimer to PI(4,5)P_2_. Incubation of 10 μM CaM with 10 μM GFP–STIM2K dimer in a buffer with 1 mM CaCl_2_ led to complex formation, which after separation by gel filtration mainly eluted in fractions 6–8 compared with elution of GFP–STIM2K dimer in fractions 7–14 and CaM in 10–14 (Supplementary Figure S4A; available at http://www.bioscirep.org/bsr/033/bsr033e077add.htm). Mutation of all four Ca^2+^-binding sites of CaM (CaM1–4m) or mutation of the Ca^2+^-binding sites in the C-terminal lobe (CaM3–4m) abolished interaction with GFP–STIM2K dimer (Figures S4B and S4C), while mutation of Ca^2+^-binding sites in the N-terminal lobe (CaM1–2m) had a milder effect (Figure S4D). This weak binding of the C-terminal lobe Ca^2+^-loaded CaM to GFP-STIM2K dimer was confirmed by elution of GFP-STIM2K with CaM1-2m from CaM beads (Figure 4B). However, compared with elution with fully Ca^2+^-loaded CaM, competition with semi Ca^2+^-loaded CaM (CaM1–2m) eluted only 21±5% of the bound GFP–STIM2K dimer compared with 45±7% with fully Ca^2+^-loaded CaM (Figure 4B). This suggests that semi- and fully Ca^2+^-load CaM bind with different affinities to STIM2 K-rich domain dimer.

### High Ca^2+^ concentration interferes with binding of STIM2 K-rich domain to PI(4,5)P_2_

**Figure 5:**
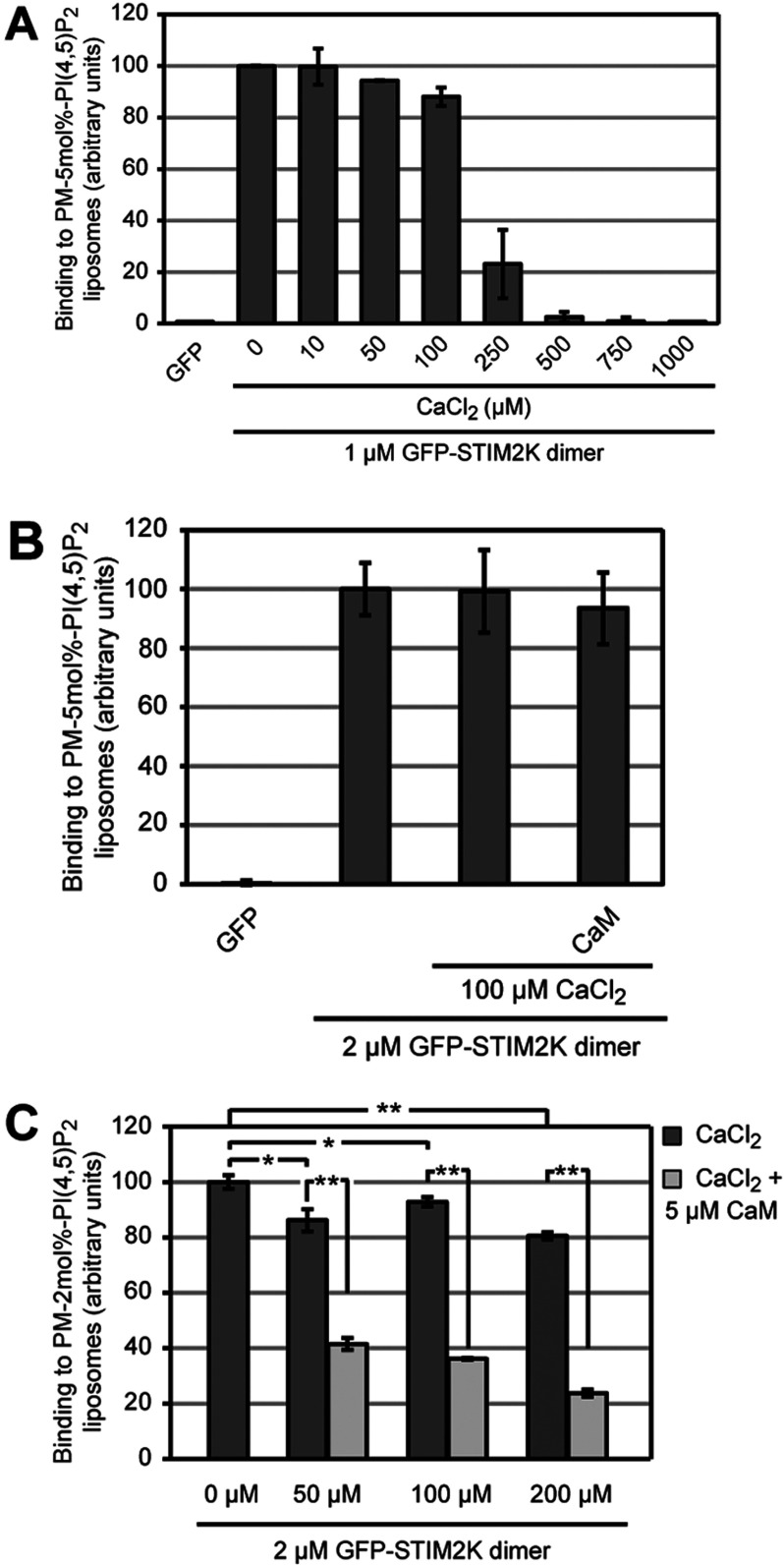
Ca^2+^ inhibits binding of STIM2 K-rich domain dimer to PI(4,5)P_2_ liposomes (**A**) Binding of 1 μM GFP and oxidized GFP-STIM2K dimer to PM-like liposomes with 5 mol% PI(4,5)P_2_ in HK buffer without Ca^2+^ and binding of GFP–STIM2K dimer in HK buffer with indicated Ca^2+^ concentrations. Binding of dimeric GFP–STIM2K in HK buffer without Ca^2+^ was set to 100. (**B**) Binding of 2 μM GFP and oxidized GFP–STIM2K dimer to PM-like liposomes with 5 mol% PI(4,5)P_2_ in HK buffer without Ca^2+^ and binding of 2 μM oxidized GFP–STIM2K and GFP-STIM2K pre-incubated for 1 h with 100 μM CaCl_2_/5 μM CaM to PM-like liposomes with 5 mol% PI(4,5)P_2_ in HK buffer with 100 μM CaCl_2_. Binding of GFP–STIM2K without CaCl_2_ and CaM was set to 100. (**C**) Binding of 2 μM GFP–STIM2K dimer to PM-like liposomes with 2 mol% PI(4,5)P_2_ in HK buffer without Ca^2+^ and binding of 2 μM dimeric GFP–STIM2K pre-incubated for 1 h with 5 μM CaM to PM-like liposomes with 2 mol% PI(4,5)P_2_ in HK buffer with 50, 100 and 200 μM CaCl_2_. Binding of GFP–STIM2K dimer in HK buffer without Ca^2+^ was set to 100 and bars indicate mean±S.D. from at least three experiments (*P<0.05, **P<0.005 and n.s. according to Student's *t* test).

Based on the binding of semi- and fully Ca^2+^-loaded CaM to the K-rich domain of STIM2, we hypothesized that high cytosolic [Ca^2+^] interferes with binding of STIM2 to PM lipids *in vivo*. This may lead to a negative-feedback regulation of STIM2-mediated ER–PM contact formation and Ca^2+^ entry. In order to investigate the effect of Ca^2+^/CaM on binding of GFP–STIM2K dimer to PM-like liposomes, we first tested whether Ca^2+^ alone has any effect on the interaction between the K-rich domain dimer and PI(4,5)P_2_. Therefore we measured the binding of 1 μM oxidized GFP–STIM2K to PM-like liposomes with 5 mol% PI(4,5)P_2_ in Ca^2+^-free buffer and set this value to 100 (Figure 5A). An increase of [Ca^2+^] up to 100 μM had no severe effect and allowed 88% binding, whereas higher [Ca^2+^] between 250 μM and 1 mM abolished the interaction completely (Figure 5A). However, when we used higher STIM2K dimer concentration the binding to 2 mol% PI(4,5)P_2_-containing liposomes was resistant to 200 μM CaCl_2_ (Figure 5C) as compared with binding of 1 μM protein in presence of 250 μM CaCl_2_ (Figure 5A). Thus, the binding of dimeric STIM2 K-rich domain to PM lipids is sensitive to high [Ca^2+^], suggesting that sustained local increase of cytosolic Ca^2+^ may interfere with STIM2-mediated ER–PM contact formation.

### Binding of Ca^2+^/CaM to STIM2 K-rich domain competes with its binding to PI(4,5)P_2_

Next, we tested the effect of Ca^2+^/CaM on binding of GFP–STIM2K dimer to PM-like liposomes with 5 mol% PI(4,5)P_2_. Pre-incubation of 2 μM GFP–STIM2K dimer with 5 μM CaM in 100 μM CaCl_2_ had no significant effect on its lipid-binding activity (Figure 5B), suggesting this amount of Ca^2+^/CaM could not compete with binding of STIM2 K-rich domain to a relative high [PI(4,5)P_2_]. Therefore we repeated this competition experiment with PM-like liposomes containing 2 mol% PI(4,5)P_2_ and tested GFP–STIM2K binding after pre-incubation with 5 μM CaM in presence of 50, 100 or 200 μM CaCl_2_. Depending on the [Ca^2+^] CaM will be either semi- or fully Ca^2+^-loaded, which may influence the degree of competition. At 100 and 200 μM CaCl_2_ the addition of CaM led to strong reduction of GFP-STIM2K binding (Figure 5C). Compared with CaCl_2_ alone, which reduced binding by 7±1 and 19±1%, the addition of CaM reduced binding by 61±2 and 71%. This corresponds to a 2.6- and 3.4-fold reduction in lipid binding by Ca^2+^/CaM at moderate [Ca^2+^]. At 50 μM CaCl_2_ the addition of CaM had a slightly smaller effect with a 2.1-fold inhibition, suggesting that the competition by CaM depends on Ca^2+^-loading of CaM and that Ca^2+^-free or semi-loaded CaM does not compete as efficient as fully Ca^2+^-loaded CaM.

**Figure 6:**
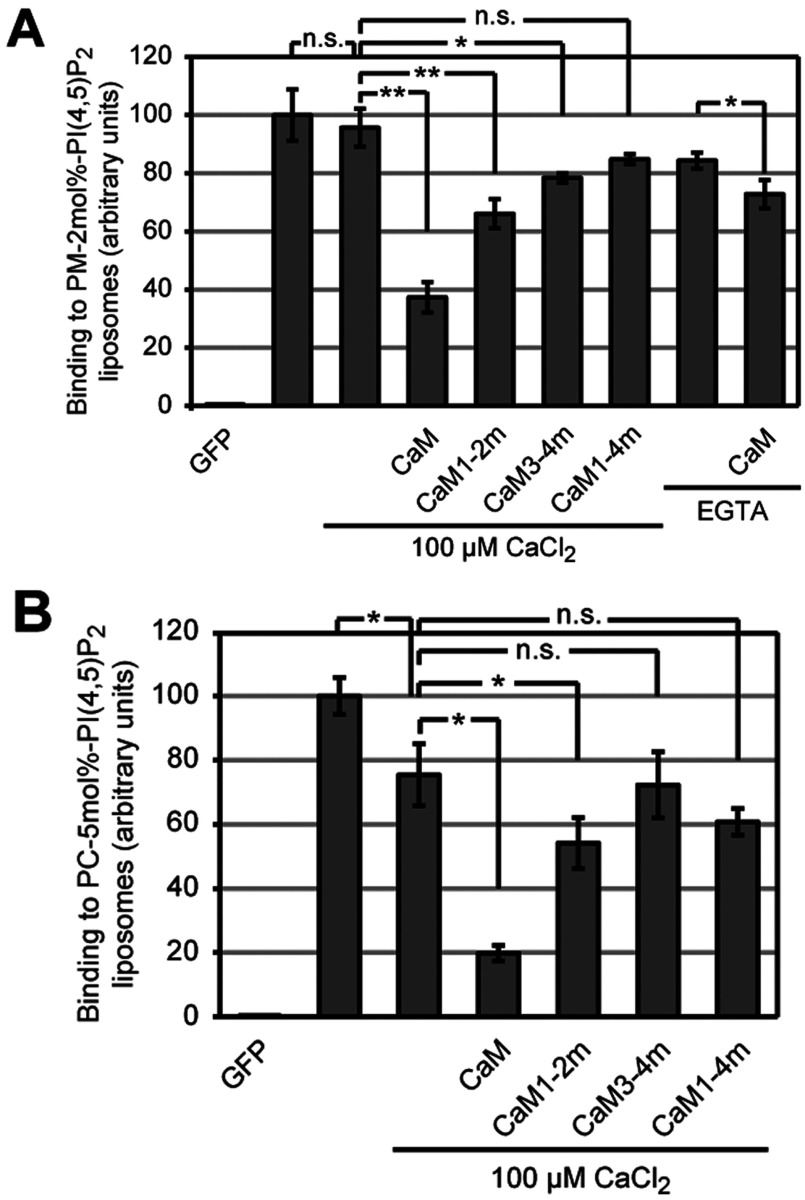
Ca^2+^/CaM competes with STIM2 K-rich domain's binding to PI(4,5)P_2_ (**A**) Binding of 2 μM reduced GFP, oxidized GFP-STIM2K dimer without Ca^2+^ and dimeric GFP–STIM2K pre-incubated with either 100 μM CaCl_2_, 5 μM CaM, CaM1–2m, CaM3–4m or CaM1–4m in presence of 100 μM CaCl_2_ to 2 mol% PI(4,5)P_2_-containing PM-like liposomes in HK buffer. Binding of dimeric GFP–STIM2K pre-incubated with either 500 μM EGTA or 500 μM EGTA/5 μM CaM to 2 mol% PI(4,5)P_2_ in HK buffer is also shown. Binding of 2 μM oxidized GFP-STIM2K dimer without Ca^2+^ to PM-like liposomes with 2 mol% PI(4,5)P_2_ in HK buffer was set to 100. (**B**) Binding of 2 μM reduced GFP, oxidized GFP–STIM2K dimer without Ca^2+^ and dimeric GFP–STIM2K pre-incubated with either 100 μM CaCl_2_, 5 μM CaM, CaM1-2m, CaM3-4m or CaM1–4m in presence of 100 μM CaCl_2_ to 5 mol% PI(4,5)P_2_-containing PC liposomes in HK buffer. Binding of 2 μM oxidized GFP–STIM2K dimer without Ca^2+^ to PC liposomes with 5 mol% PI(4,5)P_2_ in HK buffer was set to 100 and bars indicate mean±S.D. from at least three experiments (*P<0.05, **P<0.005 and n.s. according to Student's *t* test).

In order to test further whether the competition of CaM depends on Ca^2+^-loading, we pre-incubated GFP-STIM2K dimer with CaM and tested binding to PM-like liposomes with 2 mol% PI(4,5)P_2_. Compared with binding at 100 μM CaCl_2_, pre-incubation with 5 μM CaM in presence of CaCl_2_ reduced binding by 61±1% (Figure 6A). Pre-incubation of GFP–STIM2K dimer with Ca^2+^-free CaM1–4m (all EF-hand domains mutated), in presence of CaCl_2_ showed no competition, indicating that Ca^2+^-loading of CaM is required for competition with lipid binding. Consistently, CaM in presence of EGTA, only had a weak effect on lipid-binding activity of STIM2K dimer and a similar weak effect had the pre-incubation with CaM3–4m (C-terminal lobe Ca^2+^-binding mutant), suggesting that Ca^2+^-loading of the C-terminal lobe is required for competition. Mutation of the N-terminal lobe resulted in a CaM variant that showed an intermediate level of competition, i.e. 31±2% reduction in binding to 2 mol% PI(4,5)P_2_-containing PM-like liposomes. Although this effect is weaker compared with fully Ca^2+^-loaded CaM, it suggests that semi-Ca^2+^-loaded CaM at an intermediate cytosolic [Ca^2+^] may have an inhibitory effect on STIM2-mediated ER–PM contact formation.

Finally, we repeated these competition experiments with liposomes comprising only PC and 5 mol% PI(4,5)P_2_. The absence of cholesterol and sphingolipids should result in an even distribution of PI(4,5)P_2_ without formation of local clusters of PI(4,5)P_2_. Under these conditions 100 μM CaCl_2_ reduced the binding of GFP–STIM2K dimer by 25±4%, consistent with weaker binding (Figure 6B). Pre-incubation with 5 μM CaM in 100 μM CaCl_2_ reduced the binding 5-fold compared with the sample without CaCl_2_ and 3.8-fold compared with incubation with 100 μM CaCl_2_. Again, mutation of the N-lobe's EF-hand domains resulted in a CaM variant with weak competition, i.e. 28±2% reduction compared with 74±10% reduction for fully Ca^2+^-saturated CaM (Figure 6B). Mutations of the Ca^2+^-binding EF-hand domains in the C-terminal lobe or in both lobes abolished competition. In summary, these results show that Ca^2+^/CaM, local [PI(4,5)P_2_] as well as the oligomerization of STIM2 determine the binding of STIM2-containing ER to the PM lipids.

## DISCUSSION

STIM2 has evolved with a lipid-binding domain with higher affinity for PI(4,5)P_2_ than the lipid-binding domain of STIM1. In the case of STIM1, binding to PI(4,5)P_2_ requires tetramerization, whereas for STIM2 K-rich domain dimerization is sufficient for PI(4,5)P_2_ binding. The increased affinity of the STIM2 K-rich domain for PM lipids is achieved by formation of a short amphipathic α-helix. Consistently, all K-rich domains of mammalian STIM2 proteins lack a C-terminal proline residue, which is conserved in K-rich domains of STIM1. Formation of an α-helix in STIM2 K-rich domain also leads to increased affinity for Ca^2+^/CaM, which competes with binding to PI(4,5)P_2_. Thus, an elevated cytosolic [Ca^2+^] may dampen STIM2-mediated ER–PM contact formation via binding of Ca^2+^/CaM to its K-rich domain. This may contribute to a Ca^2+^-dependent feedback loop, controlling STIM2-mediated Ca^2+^ influx under conditions with increased cytosolic [Ca^2+^].

### Oligomerization controls lipid binding

Tetramerization of STIM1 K-rich domain and recognition of multiple phosphoinositide head groups provides enough binding energy for stable ER–PM contact formation. This concept of additive binding energy of individual positively charged subclusters that interact electrostatically with PI(4,5)P_2_ and PI(3,4,5)P_3_ head groups at the PM had been described previously in membrane binding studies with small GTPases [35]. Cooperation of multiple interactions is a common principle to drive multivalent membrane binding, e.g. dimerization of the dynamin PH domain increases the apparent *K*_d_ 1000-fold [36,37]. In contrast to STIM1, dimerization of the STIM2 K-rich domain and formation of an amphipathic α-helix are sufficient for binding to PM lipids. Whether STIM2 dimerization *in vivo* is sufficient for ER-PM contact formation remains open. However, overexpression of STIM2, but not STIM1 led to constitutive Ca^2+^ entry [38]. Similarly, expression of an unrelated ER protein (Kir6.2) fused to the STIM2 CTD led to constitutive ER-PM contact formation, while expression of this protein with a STIM1 CTD did not [6]. Also, whether oligomerization by oxidation of Cys^725^ in STIM2 or by any other cysteine residue in its cytosolic domain occurs *in vivo* remains open. In this context, the presence of a large number of conserved cysteine residues in the CTD of STIM2 is striking (eleven versus one cysteine in STIM1) and supports the possibility of redox-mediated STIM2 activation. Enhanced ER–PM contact formation may stimulate STIM2-dependent activation of Ca^2+^-channels, which could lead to chronic Ca^2+^ overload and finally to necrosis and cell death through apoptosis [39]. Consistent with a redox-related function of STIM2 in neuronal cells, it had been observed that STIM2^−/−^ mice fail to commit apoptosis under oxidative stress conditions [40]. However, direct evidence for redox-related control of STIM2 activity is missing. It was shown for STIM1 that redox-related control depends on its N-terminal domain in the ER lumen and oxidative stress leads to S-glutathionylation at Cys^56^ and constitutive Ca^2+^ entry [41] or to Erp57-driven intermolecular disulphide bond formation between Cys^49^ and Cys^56^ [42].

### Increased affinity of STIM2 to PM lipids and competition by Ca^2+^/CaM

Increased binding of STIM2 to PI(4,5)P_2_ could be relevant with respect to ER–PM contact formation and activation of Ca^2+^ channels in response to small changes of the ER [Ca^2+^]. The *K*_d_ of the STIM2 EF-hand domain for Ca^2+^ in the ER lumen is 0.5 mM, which will activate STIM2 at a luminal [Ca^2+^] about 0.4 mM, which is very similar to the resting [Ca^2+^] of the ER lumen [43–45]. This high K_d_ for ER Ca^2+^ and the increased binding of the STIM2 K-rich domain dimer to PI(4,5)P_2_ could contribute to the low activation threshold of STIM2, which is consistent with the previously suggested function of STIM2 in the control of Ca^2+^ homoeostasis [15]. Compared with STIM2, binding of STIM1 K-rich domain to PI(4,5)P_2_ is restricted to conditions where STIM1 proteins aggregate as a consequence of Ca^2+^ depletion from the ER lumen. The EF-hand domain of STIM1 has a *K*_d_ of 0.2 mM for the dissociation of Ca^2+^ and switching into the active form requires a more drastic drop of ER Ca^2+^ than for STIM2 [15,46]. This higher activation threshold is consistent with a function of STIM1 in ‘all or nothing’ digital signalling, where STIM1 functions as regulator of intracellular Ca^2+^ oscillations [38,47,48] or in fast repetitive Ca^2+^ release from intracellular stores in myoblasts [49]. In most cell types, low binding of monomeric or dimeric STIM1 K-rich domain to PM lipids restricts STIM1-mediated ER–PM contact formation to significant depletion of ER Ca^2+^ and avoids the activation of STIM1 by small fluctuations of the ER Ca^2+^ levels.

The evolution of the STIM2 K-rich domain into an α-helical amphipathic sequence results in enhanced PI(4,5)P_2_ and Ca^2+^/CaM binding. Depending on local [PI(4,5)P_2_] and cytosolic [Ca^2+^], Ca^2+^/CaM binding *in vivo* may compete with binding to PM lipids and thereby could help to destabilize and terminate ER–PM contacts, thereby protecting cells against Ca^2+^ overload. Consistent with a function of Ca^2+^/CaM in down-regulation of ER–PM contacts, low cytosolic [Ca^2+^] in neurons after exposure to low Ca^2+^ media enhanced STIM2–Orai1 association [50]. Together with the effect of Ca^2+^/CaM on STIM2 lipid binding, this suggests a function of Ca^2+^ store-independent ER–PM contact formation and STIM2–Orai1 interaction [51] occurring in specific cell types in addition to store-dependent STIM2–Orai1 interaction [15].

### Ca^2+^/CaM as regulator of Ca^2+^ entry

Ca^2+^/CaM is a well-established regulator of the SOCE pathway and increased cytosolic Ca^2+^ was described to disassemble STIM1 clusters [19,52]. Moreover, Ca^2+^/CaM binds Orai1 during Ca^2+^-dependent fast inactivation, where the C-terminal lobe of CaM binds to an N-terminal domain of Orai1, whereas the N-terminal lobe of CaM binds another Orai1 channel subunit [19,53]. We observed that high Ca^2+^ concentration (1 mM CaCl_2_) abolished the binding of tetrameric STIM1 C-terminus to 5 mol% PI(4,5)P_2_-containing liposomes (results not shown), suggesting that high calcium concentration interferes with lipid binding. Whether an increase in cytosolic [Ca^2+^] is sufficient to release the electrostatic interactions between STIM1 K-rich domain and PM lipids is not known but CRACR2A with two EF-hand domains stabilizes STIM1–Orai1 complexes at low cytosolic [Ca^2+^], suggesting that localized cytosolic Ca^2+^ elevations may promote the dissociation of STIM1–Orai1 complex [17]. Binding of Ca^2+^/CaM to the lipid-binding domain of STIM2 could promote such dissociation of ER–PM contacts directly and this may occur at different Ca^2+^ concentrations, since the STIM2 K-rich domain binds semi- and fully Ca^2+^-loaded CaM with different affinities. However, it remains open, whether binding of Ca^2+^/CaM to STIM2 K-rich domain can terminate an existing ER–PM contact *in vivo* or whether Ca^2+^/CaM binding has to occur before binding of STIM2 to PM lipids. The latter would inhibit the formation of new ER-PM contacts. Competition with liposome binding *in vitro* occurred only when Ca^2+^/CaM and GFP–STIM2K dimer were pre-incubated but not upon addition of Ca^2+^/CaM to liposomes bound to GFP–STIM2K dimer (results not shown).

In summary, binding of Ca^2+^/CaM to the K-rich domain of STIM2 competes with PM lipid binding and could down-regulate STIM2-mediated Ca^2+^ influx. The evolution of an overlapping lipid- and Ca^2+^/CaM-binding site in mammalian STIM2 K-rich domain allows this sensor protein to integrate information of [Ca^2+^] in the cytosol as well as in the ER lumen.

## Online data

Supplementary data
