# Supplementary material for: Oligomerization and Ca2+/calmodulin control binding of the ER Ca2+-sensors STIM1 and STIM2 to plasma membrane lipids
Source: Biosci Rep. 2013 Oct 31;33(5):e00077. doi: 10.1042/BSR20130089 (PMC3814058; doi:10.1042/BSR20130089)
Supplement: Supplementary data [file bsr033e077add.pdf]

Biosci. Rep. (2013) / **33** / art:e00077 / doi 10.1042/BSR20130089

OPEN ACCESS

Original Paper

Bioscience Reports

www.bioscirep.org

Bioscience Reports

SUPPLEMENTARY DATA

Oligomerization and Ca^{2+}/calmodulin control binding of the ER Ca^{2+}-sensors STIM1 and STIM2 to plasma membrane lipids

Rajesh BHARDWAJ*, Hans-Michael MÜLLER†, Walter NICKEL† and Matthias SEEDORF*[1]

*Zentrum für Molekulare Biologie der Universität Heidelberg (ZMBH), DKFZ-ZMBH Alliance, University of Heidelberg, Im Neuenheimer Feld 282, 69120 Heidelberg, Germany, and †Heidelberg University Biochemistry Center, Im Neuenheimer Feld 328, 69120 Heidelberg, Germany

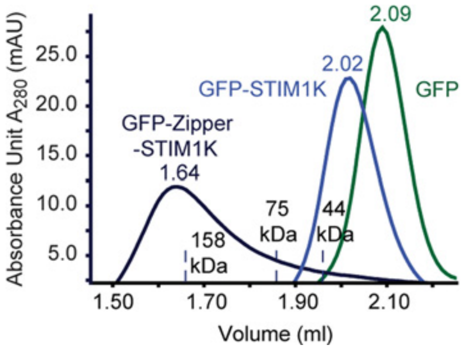

Figure S1 Addition of a leucine zipper to STIM1 K-rich domain leads to tetramerization
Elution profiles of 5 μM GFP (green), GFP–STIM1K (blue) and a GFP-tagged construct with yeast GCN4 leucine zipper fused to STIM1 K-rich domain (GFP–Zipper–STIM1K, black) S200 gel-filtration column run are shown. Positions where markers eluted are indicated.

[1] To whom correspondence should be addressed (email m.seedorf@zmbh.uni-heidelberg.de)

© 2013 The Author(s) This is an Open Access article distributed under the terms of the Creative Commons Attribution Licence (CC-BY) (http://creativecommons.org/licenses/by/3.0/) which permits unrestricted use, distribution and reproduction in any medium, provided the original work is properly cited.

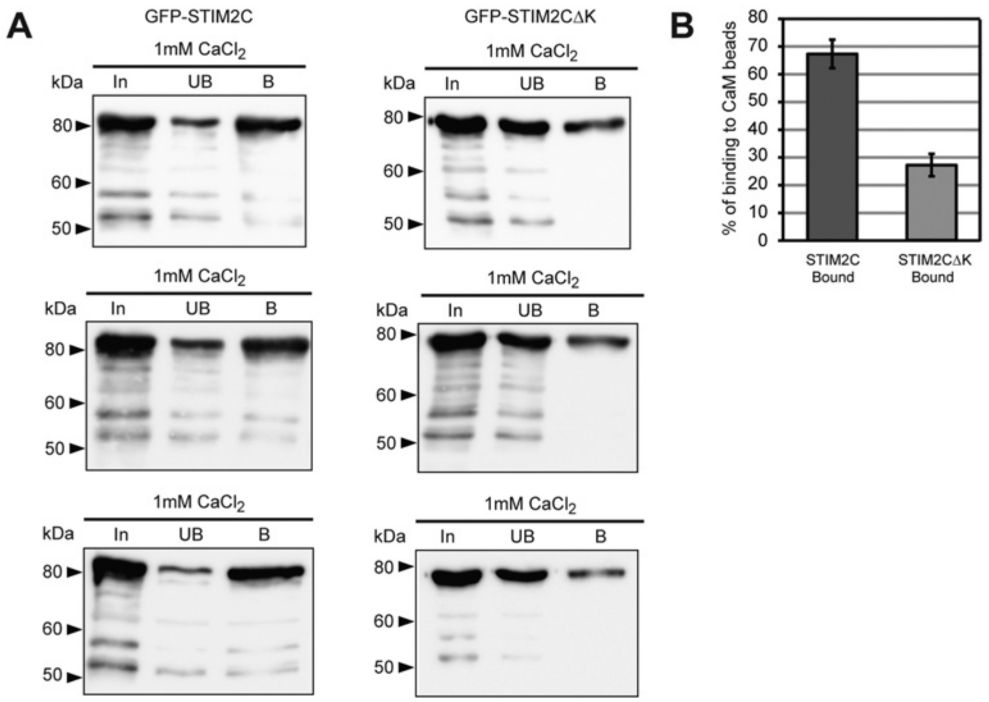

Figure S2 Western blot quantification of GFP–STIM2C and GFP–STIM2CΔK binding to Ca^{2+}/calmodulin beads
(**A**) 1 μM reduced GFP–STIM2C and GFP–STIM2CΔK were incubated with Ca^{2+}/CaM beads in presence of 1 mM $CaCl_2$. Input (In), unbound (UB) and bound (B) material of three separate experiments were detected by a rabbit polyclonal GFP antibody from Santa Cruz Biotechnology, Inc. (1:5000). (**B**) Quantification of bound GFP–STIM2C and GFP–STIM2CΔK from three experiments. Bars indicate means ± S.D.

© 2013 The Author(s) This is an Open Access article distributed under the terms of the Creative Commons Attribution Licence (CC-BY) (http://creativecommons.org/licenses/by/3.0/) which permits unrestricted use, distribution and reproduction in any medium, provided the original work is properly cited.

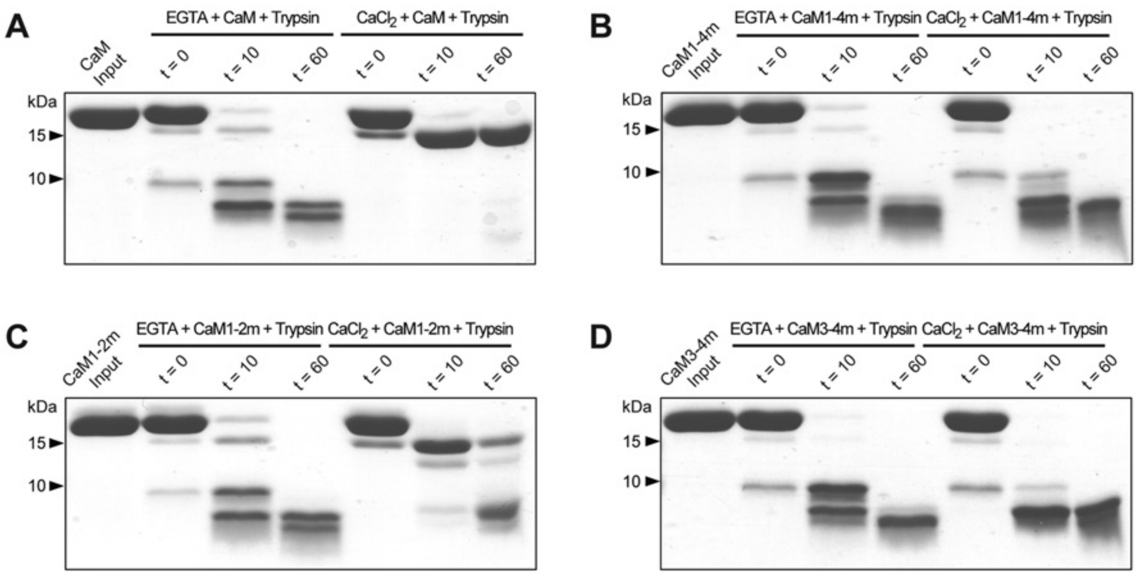

Figure S3 Protease protection assays with purified CaM and CaM mutants

14 μM CaM (**A**), CaM1–4m (**B**), CaM1–2m (**C**) and CaM3–4m (**D**) in 1 mM EGTA or 0.2 mM $CaCl_2$ were incubated with 0.02 μM trypsin in 20 mM ammonium bicarbonate buffer (pH 8.1) at 20 °C for the indicated times. The proteins were separated by SDS/PAGE and stained with Coomassie Brilliant Blue. The first lane of each gel shows the input of CaM or Ca^{2+}-binding mutants of CaM without protease.

© 2013 The Author(s) This is an Open Access article distributed under the terms of the Creative Commons Attribution Licence (CC-BY) (http://creativecommons.org/licenses/by/3.0/) which permits unrestricted use, distribution and reproduction in any medium, provided the original work is properly cited.

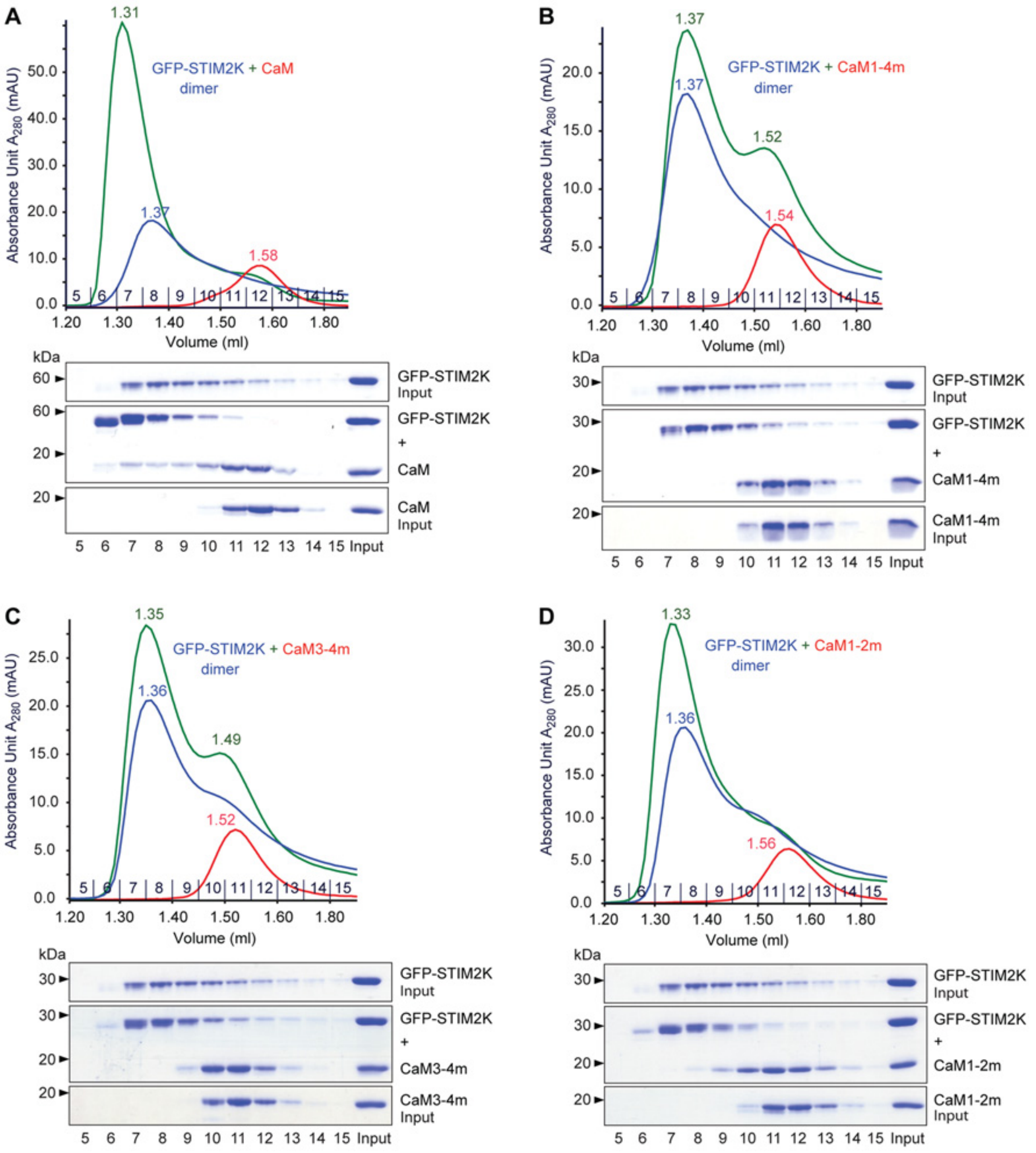

Figuere S4 In solution binding of GFP-STIM2K dimer and calmodulin
10 μM oxidized GFP–STIM2K dimer and 10 μM purified CaM (**A**), CaM1–4m (**B**), CaM3–4m (**C**) or CaM1–2m (**D**) in 1 mM $CaCl_2$ were separated by gel filtration. The elution profiles of GFP–STIM2K dimer (blue), CaM (red) and complexes (green) with indicated volume for elution peaks are shown. Proteins of the indicated eluted fractions separated by SDS/PAGE and Coomassie Brilliant Blue stained are shown below the chromatograms.

Received 12 August 2013; accepted 15 August 2013

Published as Immediate Publication 17 September 2013, doi 10.1042/BSR20130089

© 2013 The Author(s) This is an Open Access article distributed under the terms of the Creative Commons Attribution Licence (CC-BY) (http://creativecommons.org/licenses/by/3.0/) which permits unrestricted use, distribution and reproduction in any medium, provided the original work is properly cited.
